# High Throughput Screening for Natural Host Defense Peptide-Inducing Compounds as Novel Alternatives to Antibiotics

**DOI:** 10.3389/fcimb.2018.00191

**Published:** 2018-06-11

**Authors:** Wentao Lyu, Zhuo Deng, Lakshmi T. Sunkara, Sage Becker, Kelsy Robinson, Robert Matts, Guolong Zhang

**Affiliations:** ^1^Department of Animal Science, Oklahoma State University, Stillwater, OK, United States; ^2^Department of Biochemistry and Molecular Biology, Oklahoma State University, Stillwater, OK, United States; ^3^Department of Physiological Sciences, Center for Veterinary Health Sciences, Oklahoma State University, Stillwater, OK, United States

**Keywords:** host defense peptides, antimicrobial peptides, defensins, high throughput screening, HDP inducers, wortmannin, host-directed antimicrobial therapy, antimicrobial resistance

## Abstract

A rise in antimicrobial resistance demands novel alternatives to antimicrobials for disease control and prevention. As an important component of innate immunity, host defense peptides (HDPs) are capable of killing a broad spectrum of pathogens and modulating a range of host immune responses. Enhancing the synthesis of endogenous HDPs has emerged as a novel host-directed antimicrobial therapeutic strategy. To facilitate the identification of natural products with a strong capacity to induce HDP synthesis, a stable macrophage cell line expressing a luciferase reporter gene driven by a 2-Kb avian β-defensin 9 (*AvBD9*) gene promoter was constructed through lentiviral transduction and puromycin selection. A high throughput screening assay was subsequently developed using the stable reporter cell line to screen a library of 584 natural products. A total of 21 compounds with a minimum Z-score of 2.0 were identified. Secondary screening in chicken HTC macrophages and jejunal explants further validated most compounds with a potent HDP-inducing activity in a dose-dependent manner. A follow-up oral administration of a lead natural compound, wortmannin, confirmed its capacity to enhance the *AvBD9* gene expression in the duodenum of chickens. Besides *AvBD9*, most other chicken HDP genes were also induced by wortmannin. Additionally, butyrate was also found to synergize with wortmannin and several other newly-identified compounds in *AvBD9* induction in HTC cells. Furthermore, wortmannin acted synergistically with butyrate in augmenting the antibacterial activity of chicken monocytes. Therefore, these natural HDP-inducing products may have the potential to be developed individually or in combinations as novel antibiotic alternatives for disease control and prevention in poultry and possibly other animal species including humans.

## Introduction

Antimicrobial resistance is posing a major threat to public health. While it is necessary to continue the development of antibiotics with direct antimicrobial activities, host-directed therapies have emerged as attractive alternative strategies to combating infectious and non-communicable diseases (Zumla et al., 2016). Host defense peptides (HDPs), also known as antimicrobial peptides, are represented by a large diverse group of small peptides that are synthesized primarily by phagocytic cells and epithelial cells lining the gastrointestinal, respiratory, and urogenital tracts (Zasloff, 2002). With antimicrobial, immunomodulatory and barrier protective activities, HDPs constitute an important phylogenetically conserved first line of defense in virtually all species of life (Hilchie et al., 2013; Mansour et al., 2014; Robinson et al., 2015). Two main HDP families, namely defensins and cathelicidins, exist in vertebrate animals (Zanetti, 2004; Selsted and Ouellette, 2005). One cathelicidin known as LL-37, six α-defensins, and a minimum of 39 β-defensins exist in humans (Wang, 2014), whereas four cathelicidins (CATH1-3 and CATH-B1) and 14 avian β-defensins (AvBD1-14) are present in chickens (Cuperus et al., 2013; Zhang and Sunkara, 2014).

While HDPs are being directly explored as novel antimicrobials or vaccine adjuvants against drug-resistant infections (Choi et al., 2012; Hilchie et al., 2013; Mansour et al., 2014), modulating the synthesis of endogenous HDPs has shown promise in the treatment of shigellosis, pulmonary tuberculosis, cholera, and enteropathogenic *E. coli*-induced diarrhea (Raqib et al., 2012; Al-Mamun et al., 2013; Mily et al., 2015; Sarker et al., 2017). In fact, a number of small-molecule compounds such as butyrate, vitamin D_3_, bile acids, and histone deacetylase inhibitors have been shown to induce HDP synthesis in humans without provoking inflammation (Campbell et al., 2012; Van Der Does et al., 2012; Lyu et al., 2015; Yedery and Jerse, 2015). A high throughput screening (HTS) luciferase reporter assay was recently developed to identify multiple compounds with the ability to induce human *LL-37* gene expression (Nylen et al., 2014).

To facilitate the identification of HDP-inducing compounds for use in other animal species, particularly in poultry, here we report the establishment of a cell-based HTS assay. Our earlier studies revealed that, among multiple chicken HDPs, *AvBD9* is the most readily inducible gene in response to butyrate and several other compounds in chickens (Sunkara et al., 2011, 2014). Here we constructed a stable chicken macrophage cell line integrated permanently with a lentiviral luciferase reporter vector under control of chicken *AvBD9* gene promoter. Such a stable cell line was further employed to screen a library of 584 natural products. Multiple *AvBD9*-inducing compounds were identified and further validated for their HDP-inducing activities *in vitro, ex vivo*, and *in vivo*. We confirmed several natural compounds such as wortmannin to have a strong ability to enhance HDP gene expression with good potential for further development as novel antibiotic alternatives for application in poultry and possibly other animal species.

## Materials and methods

### Chemicals

Cell culture media and supplements such as RPMI 1640, DMEM, PBS, and antibiotics (penicillin, streptomycin, and puromycin) were purchased from Lonza (Allendale, NJ), Fisher Scientific (Pittsburgh, PA) or Santa Cruz Biotechnology (Dallas, TX). Heat-inactivated fetal bovine serum (FBS) was obtained from Atlanta Biologicals (Flowery Branch, GA). Sodium butyrate and sanguinarine were procured from Sigma-Aldrich (St. Louis, MO). Trichostatin A (TSA), apicidin, HC toxin, LY294002, PX866, CAL-101, MK2206, Triciribine, GDC0068, Rapamycin, AZD8055, and BEZ235 were obtained from Cayman Chemical (Ann Arbor, MI). Tetrandrine was acquired from Santa Cruz Biotechnology, and (–)-depudecin was purchased from BioVision (Milpitas, CA) and MyBioSource (San Diego, CA). Datiscetin was ordered from BOC Sciences (Shirley, NY), while wortmannin and CUDC-907 were procured from Selleck Chemicals (Houston, TX).

### Cell culture

Chicken HTC macrophage cells (Rath et al., 2003), kindly provided by Dr. Narayan C. Rath of USDA-ARS, were cultured in RPMI 1640 containing 10% heat-inactivated FBS, 100 U/ml penicillin, and 100 μg/ml streptomycin. Stable HTC cell lines (HTC/*AvBD9*-*luc*) transduced with the *AvBD9-*driven luciferase gene were maintained in the same complete medium supplemented additionally with 0.5 μg/ml puromycin. Human 293T embryonic kidney epithelial cells (HEK 293T) were obtained from ATCC (Manassas, VA) and cultured in DMEM containing 10% FBS, 100 U/ml penicillin, and 100 μg/ml streptomycin. All cells were maintained at 37°C and 5% CO_2_ and subcultured every 3–4 days.

### Construction of the *AvBD9* luciferase reporter plasmids

Chicken genomic DNA was extracted from the liver of a Cobb broiler chicken using Quick-gDNA Microprep Kit (Zymo Research, Irvine, CA) according to the manufacturer's recommendations. A series of *AvBD9* gene promoter constructs were cloned from chicken genomic DNA using CloneAmp HiFi PCR Premix (Takara Bio USA, Mountain View, CA) with different forward primers paired with a common reverse primer (Table 1). It is noted that the 5′-end of gene-specific reverse primer begins at the third nucleotide upstream of the start codon of the *AvBD9* mRNA (GenBank accession number NM_001001611). PCR products were then cloned into a *Kpn*I-linearized luciferase reporter vector, pGL4.21[*luc2P*/Puro] (Promega, Madison, WI), using a ligation-independent In-Fusion HD PCR Cloning Kit (Takara Bio USA). The presence of the insert in each recombinant plasmid was confirmed with direct Sanger sequencing. Recombinant plasmids were propagated in Stellar *E. coli* HST08 competent cells (Takara Bio USA) and purified with QIAprep Spin Plasmid Miniprep Kit (Qiagen, Germantown, MD) for transient transfections as described below.

**Table 1:** Primers used in this study^a^^,^^b^.

**Name**	**Sequence**	**Size (bp)**
**AvBD9 FORWARD PRIMERS**		
AvBD9-120-F	TGGCCTAACTGGCCGGTACCGTCCAGACCCACAGCCTTTA	118
AvBD9-300-F	TGGCCTAACTGGCCGGTACCTCTCTGGGTGCAGCCCA	298
AvBD9-399-F	TGGCCTAACTGGCCGGTACCCAACACCATGTCCAAGAGCCAC	397
AvBD9-611-F	TGGCCTAACTGGCCGGTACCAGATATCAAGGACAGGGATGGG	609
AvBD9-950-F	TGGCCTAACTGGCCGGTACCCCTCAAGAGTGGCATTTCTCAG	948
AvBD9-1999-F	TGGCCTAACTGGCCGGTACCGTGGATGCTGTTATTGCCTGGA	1,997
AvBD9-2998-F	TGGCCTAACTGGCCGGTACCGAGATCTGCAGGAAAGCAGCT	2,996
AvBD9-3948-F	TGGCCTAACTGGCCGGTACCAAACAGGAATTTCCACATGGCAG	3,946
AvBD9-1999-Lenti-F	TTTTATCGATGAATTCGTGGATGCTGTTATTGCCTGGA	1,997
**AvBD9 REVERSE PRIMERS**		
AvBD9-3-R	CCGGATTGCCAAGCTTTTGTCCTCTGCTGTGGAATAG	
AvBD9-3-Lenti-R	TACACGCCTAACTAGTTTGTCCTCTGCTGTGGAATAG	

a *Each forward primer consists of a common linker sequence at the 5′-end and a KpnI site in the middle (as underlined) and a gene-specific sequence at the 3′-end, whereas the reverse primer included a different linker sequence at the 5′-end and a HindIII site in the middle (as underlined) and a gene-specific sequence at the 3′-end. The exceptions are AvBD9-1999-Lenti-F and AvBD9-3-Lenti-R that are composed of different linker sequences and restriction sites*.

b *The number associated with each primer indicates the upstream position relative to the start codon of the AvBD9 mRNA reference sequence (GenBank accession no. NM_001001611)*.

### Transient transfection and luciferase assay

HTC cells were seeded overnight in 24-well tissue culture plates before being transfected with 50 ng/well of different *AvBD9* promoter-driven luciferase reporter plasmids using FuGENE HD Transfection Reagent (Promega). After 24 h, cells were stimulated in duplicate with or without 8 mM sodium butyrate for another 24 h. Luciferase activity was measured by adding an equal volume of Steady-Glo Substrate to each well for 10 min using Steady-Glo Luciferase Assay System (Promega) according to the manufacturers' instructions. The luminescence was detected using Modulus Single-Tube Luminometer (Turner Biosystems, Sunnuvale, CA).

### Development of a stable HTC/*AvBD9-luc* luciferase reporter cell line

A 2.0-Kb *AvBD9* gene promoter fragment was cloned into a lentiviral luciferase reporter vector, pGreenFire1-mCMV-Puro (System Biosciences, Palo Alto, CA) using In-Fusion HD PCR Cloning Kit (Takara Bio USA) and gene-specific primers (Table 1). The PCR product in the recombinant plasmid was confirmed by Sanger sequencing. Pseudolentiviral particles were packaged by transfecting HEK 293T cells in a 10-cm tissue culture dish with 1 μg of recombinant *AvBD9* reporter lentivector and 5 μg of the pPACKH1 plasmid mix (System Biosciences) using Lipofectamine 3000 Reagent (Thermo Fisher Scientific) according to the manufacturer's instructions. The cell culture medium containing pseudolentiviral particles was collected 48 h after transfection and stored at −80°C. For viral transduction, HTC cells were seeded at 1 × 10^5^ cells/well in a 6-well plate overnight and then incubated with 2 ml HEK 293T cell culture medium containing the pseudolentiviruses for 4 h before being replenished with 4 ml fresh cell culture medium. After 3 days of incubation, transduced HTC cells were expanded to 10-cm dishes in complete RPMI 1640 medium containing 0.5 μg/ml puromycin for a week of selection, with medium change every 2–3 days. Single cell clones were obtained by limiting dilution of stable cells in 96-well plates in complete RPMI 1640 medium in the presence of 0.5 μg/ml puromycin. After 10–14 days, individual cell clones were gradually expanded and assessed for their responsiveness to sodium butyrate. The most responsive cell clones, named HTC/*AvBD9-luc*, were chosen for the development of a high-throughput screening (HTS) assay.

### Optimization of a cell-based HTS assay for *AvBD9*-lnducing compounds

Stable HTC/*AvBD9-luc* cells were grown overnight at different densities in the presence or absence of FBS in a 96-well white tissue culture plate with clear bottom (Santa Cruz Biotechnology). Cells were stimulated with 8 mM butyrate for 24 h, followed by luminescence detection with Steady-Glo Luciferase Assay System (Promega) on L-Max II Luminescence Microplate Reader (Molecular Devices, Sunnyvale, CA). To assess the robustness of the HTS assay, Z'-factor (Zhang et al., 1999) was used, which is expressed as Z'$=1-\frac{\left(3\sigma_{p}+3\sigma_{n}\right)}{\left|\mu_{p}-\mu_{n}\right|}$, where σ_*p*_ and σ_*n*_ are standard deviations of positive and negative controls, while μ_*p*_ and μ_*n*_ are the mean luciferase activity of positive and negative controls, respectively. To assess the Z'-factor, HTC/*AvBD9-luc* cells were grown at 4 × 10^4^ cells/well overnight in 96-well white plates in 50 μl complete RPMI 1640 medium, followed by stimulated with or without 8 mM butyrate in 48 technical replicates for another 24 h. Luciferase activity was measured with Steady-Glo Luciferase Assay System on L-Max II Luminescence Microplate Reader.

### Screening of natural product libraries

HTC/*AvBD*9-*luc* cells were seeded at 4 × 10^4^ cells/well overnight in 96-well white tissue culture plates in complete RPMI 1640 medium containing 0.5 μg/ml puromycin. The natural products and rare natural products libraries consisting of 584 compounds were previously purchased from BIOMOL International (Plymouth Meeting, PA) (Davenport et al., 2014), dissolved in DMSO at 10 mg/ml, and further diluted in RPMI 1640–0.2 mg/ml. Compounds were then added to individual wells to a final concentration of 20 μg/ml for 24 h, followed by luciferase assay with Steady-Glo Luciferase Assay System (Promega) on L-Max II Luminescence Microplate Reader (Molecular Devices). Cell viabilities were also assessed by adding alamarBlue Reagent (Thermo Fisher Scientific, Waltham, MA) to cell culture to a final concentration of 0.2% 4 h before luciferase assay. Fluorescence was detected on FLx800 Multi-Detection Microplate Reader (BioTek, Winooski, VT) at the excitation/emission wavelengths of 570 nm and 590 nm, respectively. The relative luciferase activity was determined for each compound after normalization to the cell viability. For selection of positive compounds, Z-score (Curtis et al., 2016) was calculated, which is defined as Z $=\frac{x-\mu }{\sigma }$, where x is relative luciferase activity of an individual compound, μ is the mean luciferase activity of all test compounds, and σ is the standard deviation of all test compounds in a 96-well plate. A compound with a minimum Z-score of 2.0, meaning that its luciferase activity is two standard deviations above that of the mean, was considered a hit (Curtis et al., 2016).

### Secondary screening of the hit compounds

Dose-response experiments were conducted in 96-well plates seeded with HTC/*AvBD9-luc* cells and treated with three different concentrations (5, 20, and 80 μg/ml) of all hits in duplicate for 24 h. Cell viability and luciferase assays were conducted as described above. For those compounds showing a robust dose-dependent response, their HDP-inducing activities were further validated in parental HTC cells (6 × 10^5^/well) at different concentrations in 12-well plates. After 24 h stimulation, cells were subjected to RNA isolation and real-time RT-qPCR as described below.

### RNA extraction and RT-qPCR

After stimulation, cells were directly lysed in RNAzol RT (Molecular Research Center, Cincinnati, OH), followed by total RNA extraction. Maxima First-Strand cDNA Synthesis Kit (Thermo Fisher Scientific) or iSCRIPT RT Supermix (Bio-Rad) was used for cDNA synthesis and qPCR was performed using QuantiTect SYBR Green qPCR Master Mix (Qiagen, Valencia, CA) or iTaq Universal SYBR Green Supermix (Bio-Rad) as described (Sunkara et al., 2011, 2012, 2014). The expression levels of various chicken HDP genes as well as a house-keeping gene, glyceraldehyde-3-phosphatedehydrogenase (*GAPDH*), were evaluated using gene-specific primers, and relative fold changes in gene expression were calculated using the ΔΔCt method as described (Sunkara et al., 2011, 2012, 2014).

### Intestinal explant culture

Chicken jejunal explants were prepared as described (Sunkara et al., 2014). Briefly, an approximately 10-cm jejunal segment was collected from 1- to 2-week-old broiler chickens, washed thoroughly in cold PBS containing 100 μg/ml of gentamicin, 100 U/ml penicillin, and 100 μg/ml streptomycin, and dissected into a series of small segments (approximately 5 × 5 mm). Jejunal segments were then placed individually in 6-well plates containing 4 ml RPMI 1640 medium supplemented with 10% FBS, 20 mM HEPES, 100 μg/ml gentamicin, 100 U/ml penicillin, and 100 μg/ml streptomycin. The segments were treated in triplicate with different concentrations of a compound and then incubated in a Hypoxia Chamber (StemCell Technologies, Vancouver, BC, Canada) flushed with 95% O_2_ and 5% CO_2_ at 37°C for 24 h. Total RNA isolation and RT-qPCR analysis of chicken HDP gene expression were performed with jejunal explants after stimulation.

### Oral gavage of HDP-inducing compounds to chickens

A total of 72 newly hatched male broiler chickens were obtained from Cobb-Vantress Hatchery (Siloam Springs, AR), housed on floor cages, divided randomly into groups of 6, and provided *ad libitum* access to a commercial antibiotic-free diet (DuMOR Chick Starter/Grower 20%) and tap water. After 3 days of acclimation, for each treatment group, 12 chickens in two cages were orally gavaged every 12 h for three times with 0.5 ml of PBS alone or PBS containing 5, 10, 20, or 40 μM wortmannin or 40 mM sodium butyrate. After 36 h of initial gavage, all birds were euthanized with carbon dioxide and cervically dislocated. A segment of the mid-duodenum was collected, snap frozen in liquid nitrogen, and stored at −80°C for future homogenization in RNAzol RT and RNA extraction. This study was carried out in accordance with the recommendations of the Guide for the Care and Use of Agricultural Animals in Research and Teaching, 3rd edition (2010), by Federation of Animal Science Societies. The protocol was approved by the Institutional Animal Care and Use Committee of Oklahoma State University under protocol number AG1610.

### Minimum inhibitory concentration (MIC) assay

The MICs of wortmannin and butyrate were determined using a standard broth microdilution assay as recommended by the Clinical and Laboratory Standards Institute (National Committee for Clinical Laboratory Standards, 2003) as we previously described (Xiao et al., 2006, 2009; Bommineni et al., 2007). Briefly, *Salmonella enterica* subsp. *enterica* serovar Enteritidis (ATCC 13076) and *Escherichia coli* (ATCC 25922) were streaked onto trypticase soy agar (Fisher Scientific) plates, followed by subculture of 2–3 individual colonies in trypticase soy broth (Fisher Scientific) with shaking at 37°C for 3 h to reach the mid-log phase. Bacteria were then diluted to 5 × 10^5^ CFU/ml in Mueller Hinton Broth (Fisher Scientific). After dispensing 90 μl/well in a 96-well tissue culture plate, 10 μl of wortmannin were added in duplicate to final concentrations of 5, 10, 20, 40, 80, 160, and 320 μM with or without 4 mM sodium butyrate. MIC was determined as the lowest concentration of the compound or compound combination that gave no visible bacterial growth after overnight incubation at 37°C.

### Antimicrobial activity of chicken monocytes

The antibacterial activities of chicken monocytes treated with wortmannin, butyrate or their combination was assessed as described previously (Schauber et al., 2006; Sunkara et al., 2011) with slight modifications. In brief, peripheral blood mononuclear cells (PBMCs) were isolated from EDTA-anticoagulated venous blood of 1- to 4-week-old male Cobb broilers through gradient centrifugation using Histopaque 1077 (Sigma-Aldrich). Monocytes were obtained by seeding PBMCs at 3 × 10^7^ cells/well in complete RPMI 1640 medium containing 10% FBS, 20 mM HEPES, 100 U/ml penicillin, and 100 mg/ml streptomycin in 6-well plates overnight and washing off non-adherent cells twice with calcium- and magnesium-free Hank's balanced salt solution (HyClone, Pittsburgh, PA). Monocytes were replenished with fresh complete RPMI 1640 medium and stimulated in duplicate with 40 μM wortmannin in the presence or absence of 4 mM butyrate. After 24 h, cells were scraped, washed twice with calcium- and magnesium-free Hank's balanced salt solution, and resuspended in 100 μl water. Cells were then frozen at −80°C for 20 min, thawed, and sonicated for 30 s, followed by centrifugation at 12,000 × *g* for 10 min at 4°C. Cell supernatants were collected and 20 μl of the supernatants were incubated with 80 μl of *S. enteritidis* (ATCC 13076) at 2.5 × 10^5^ CFU/ml in 20% trypticase soy broth containing 1 mM NaH_2_PO_4_ and 25 mM NaHCO_3_ in a 96-well plate at 37°C. Bacterial turbidity was measured at OD_600_ using SpectraMax M3 (Molecular Devices, Sunnyvale, CA) at 3, 6, 9, and 24 h.

### Statistical analysis

Data are expressed as means ± SEM. Statistics was performed with GraphPad Prism (San Diego, CA) using unpaired Student's two-tailed *t*-test. *P* < 0.05 was considered significant.

## Results

### Selection of appropriate *AvBD9* gene promoter constructs to establish a stable cell line

**Figure 1:**
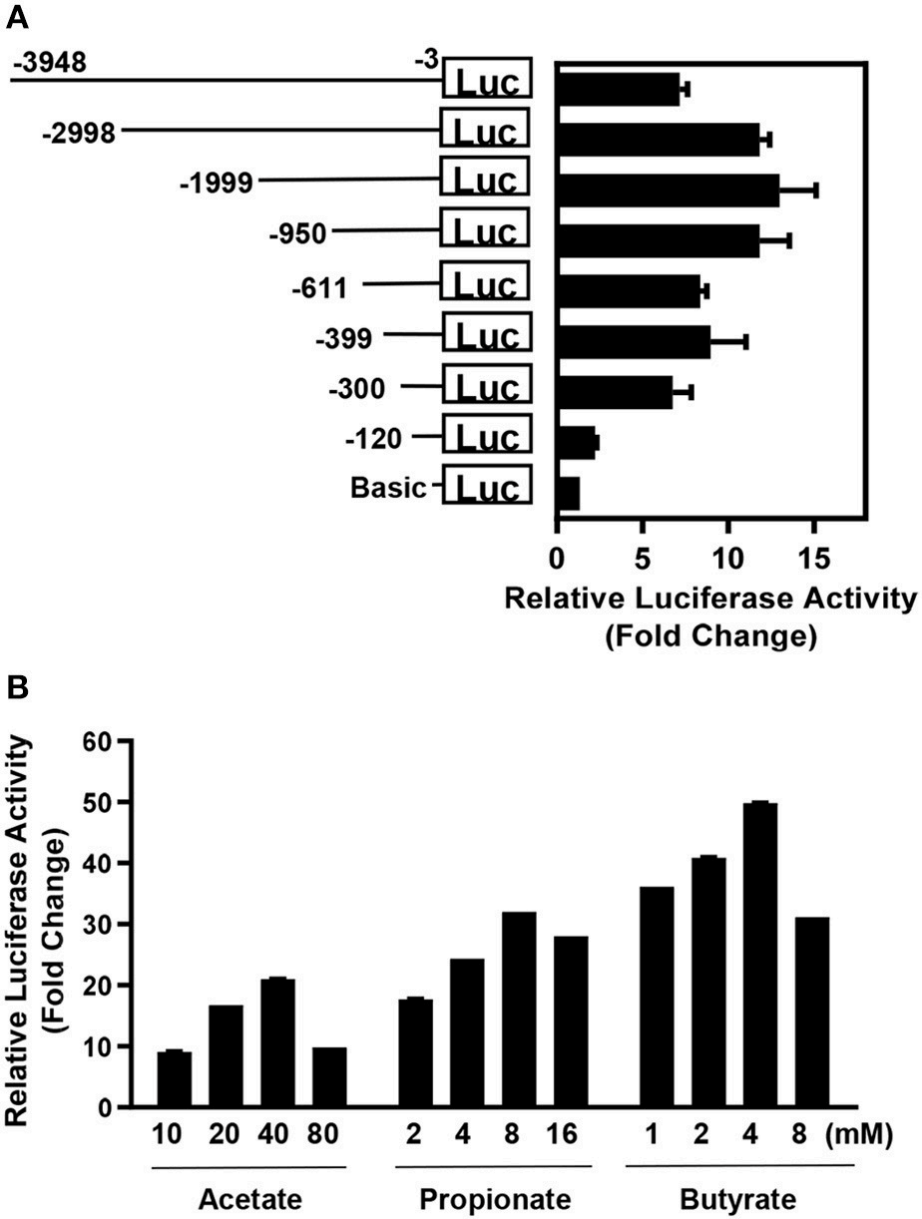
Promoter analysis of the *AvBD9* gene. **(A)** *AvBD9* gene promoter constructs of different lengths were cloned into pGL4.21[*luc2P*/Puro] luciferase reporter vector and transfected into chicken HTC macrophage cells, followed by stimulation with 8 mM sodium butyrate for 24 h. The fold change in luciferase activity of the cells transfected with each *AvBD9* promoter construct in response to butyrate relative to that of the cells transfected with the promoterless basic vector is shown. **(B)** Chicken HTC cells were transfected with the pGL4.21[*luc2P*/Puro] luciferase reporter vector driven by a 2-Kb *AvBD9* gene promoter construct, followed by stimulation with or without different concentrations of short-chain fatty acids for 24 h. For each short-chain fatty acid, fold change in luciferase activity of stimulated cells was calculated relative to that of the non-stimulation control. The results are means ± SEM of 2–3 independent experiments.

*AvBD9* has been shown to be the most inducible HDP gene in response to butyrate and several other small-molecule compounds in chickens (Sunkara et al., 2011, 2014). Therefore, the *AvBD9* gene promoter was chosen to drive luciferase reporter gene expression. In order to select an appropriate promoter segment to provide maximum luciferase activation, eight *AvBD9* promoter constructs of varying lengths were cloned into a luciferase reporter vector, pGL4.21[*luc2P*/Puro] (Promega). The recombinant vectors were separately transfected into chicken HTC macrophage cells and stimulated with 8 mM sodium butyrate for 24 h. Luciferase assay revealed that *AvBD9* promoter activity was clearly length-dependent, with the 2-Kb promotor construct giving a maximum 13-fold increase in luciferase activity relative to a basic promoterless construct (Figure 1A). Consequently, the 2-Kb *AvBD9* construct was used for subsequent stable cell line development. Besides butyrate, the 2-Kb *AvBD9* construct was also confirmed to respond to two other short-chain fatty acids, namely acetate and propionate, in a dose-dependent manner after transfection into HTC cells (Figure 1B).

It is noted that omission of either of two promoter regions (120–300 or 611–950 bp) upstream of the *AvBD9* start codon resulted in greatly diminished luciferase activity in response to butyrate (Figure 1A), implying the presence of consensus binding sites for critical transcription factors in these two regions. Conversely, inclusion of a 950-bp segment upstream of the 2,998-bp region obviously suppressed the luciferase activity (Figure 1A), suggesting the existence of the binding site for a negative regulator. A preliminary scanning for putative transcription factor binding sites in those regions revealed several candidate transcription factors (data not shown), which are currently being experimentally verified.

### Establishment of a cell-based HTS assay to identify *AvBD9*-inducing compounds

**Figure 2:**
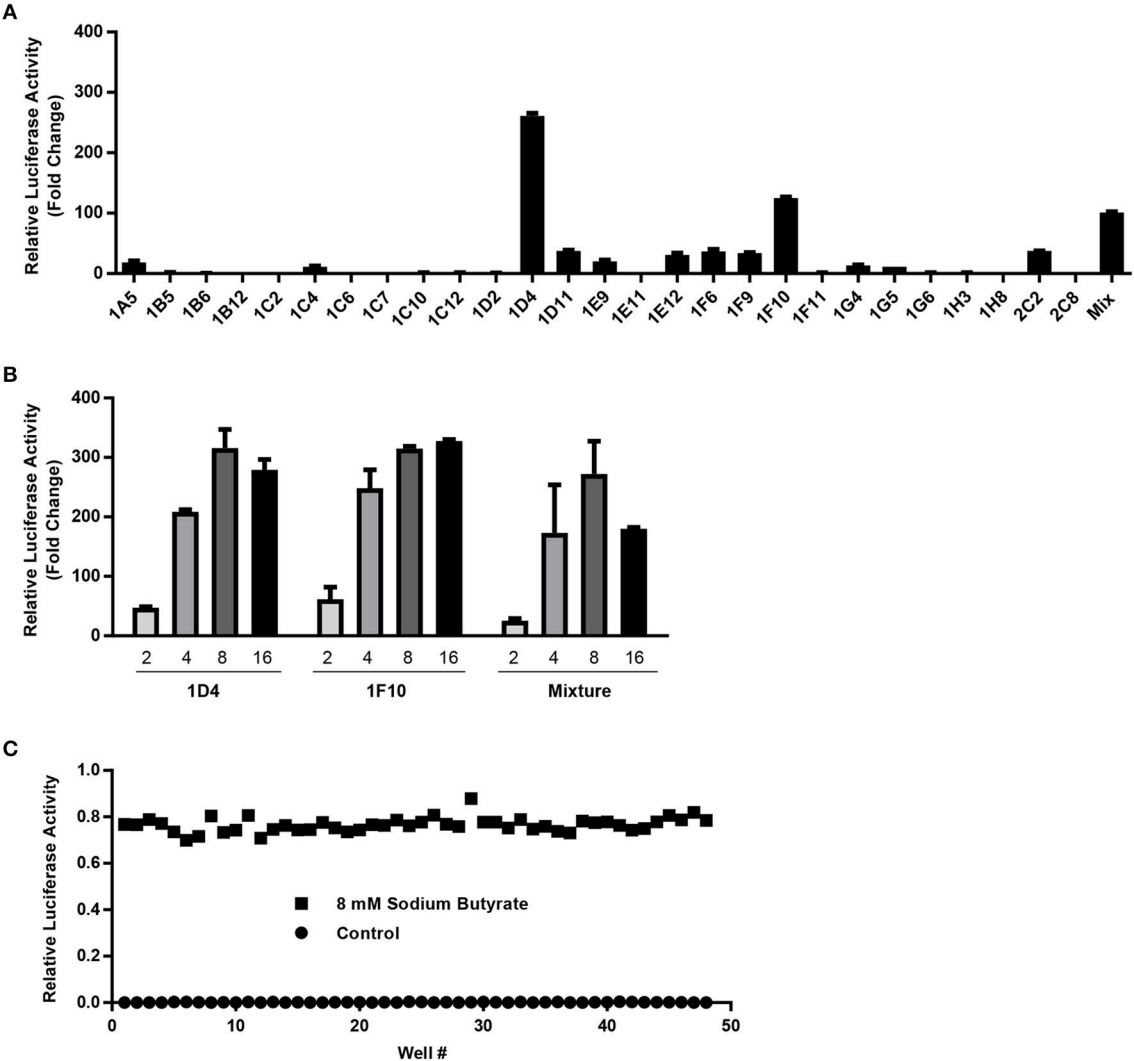
Characterization and optimization of stable HTC/*AvBD9-luc* luciferase reporter cells for high-throughput screening. **(A)** The fold change in luciferase activity of each cell clone in response to 8 mM butyrate relative to that of non-stimulation control. **(B)** Dose-dependent response of two selected stable cell clones and stable cell mixture to sodium butyrate (mM). The results in **(A,B)** are means ± SEM of two independent experiments. **(C)** Relative luciferase activities of stable reporter cells in the presence or absence of 8 mM sodium butyrate for calculation of the Z'-factor.

To establish a stable luciferase reporter cell line driven by the *AvBD9* gene promoter, the 2-Kb *AvBD9* promoter construct that gave the highest fold increase in response to butyrate (Figure 1A) was cloned into a lentiviral luciferase reporter vector, pGreenFire1-mCMV-Puro (System Biosciences). Pseudoviruses were generated in 293T cells and subsequently used to infect chicken HTC macrophages. After 1 week of selection in puromycin, a portion of surviving cells were subjected to limiting dilution in 96-well plates. Individual cell clones were gradually expanded, followed by evaluation of their responsiveness to butyrate. Among 27 cell clones analyzed, 1D4 and 1F10 showed the highest fold increase, and both were superior to the mixture of cells prior to limiting dilution (Figure 2A). These two cell clones were further confirmed to contain the 2-Kb transgene by PCR (data not shown) and gave a similar 300-fold increase in luciferase activity following 24-h stimulation with 8 mM sodium butyrate (Figure 2B). Therefore, these two stable reporter cell clones, named HTC/*AvBD9-luc*, were used interchangeably and 8 mM butyrate was used as positive control in subsequent HTS assays. To further evaluate the robustness of the HTS assay, we assessed the Z'-factor (Zhang et al., 1999) by measuring luciferase activity of stable cells stimulated with or without 8 mM butyrate in a 96-well plate. Positive controls (8 mM butyrate) were clearly separated from negative controls (no stimulation) (Figure 2C), and the Z'-factor was calculated to be 0.80, indicating that the HTS assay is excellent (Zhang et al., 1999).

### Identification and validation of natural HDP-inducing compounds

**Figure 3:**
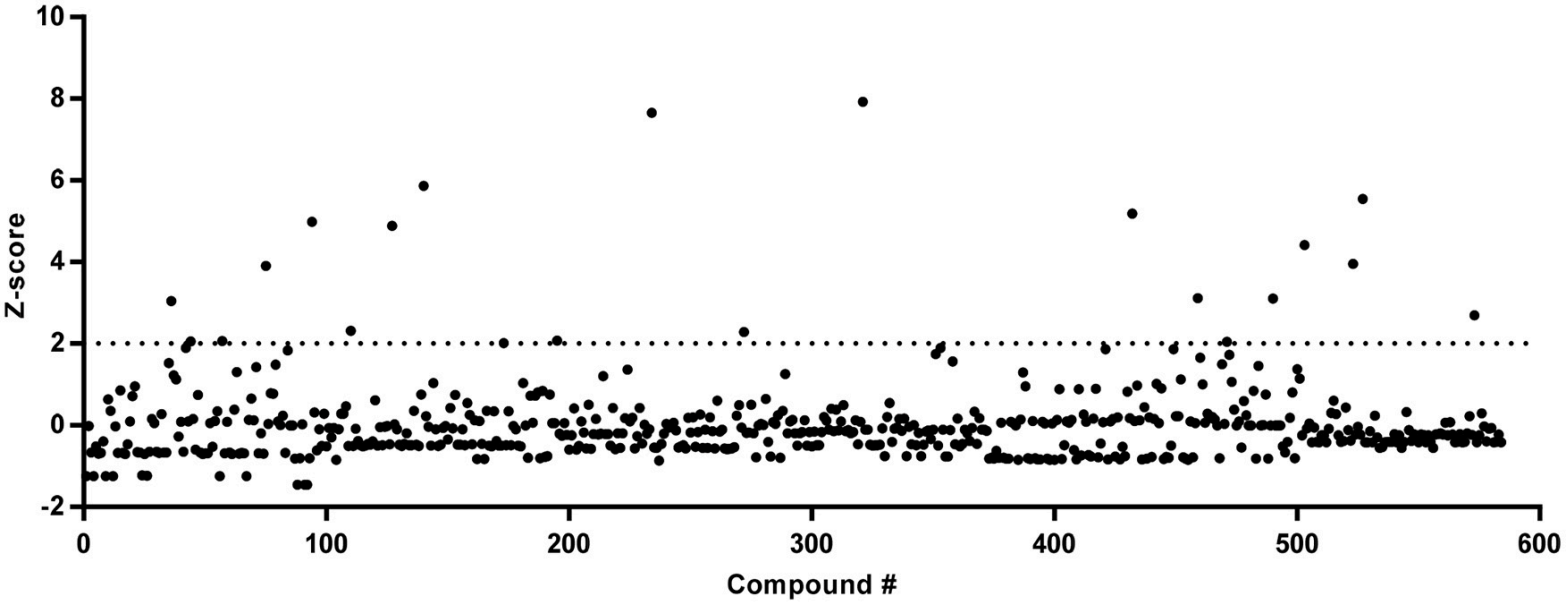
Z-scores of 584 natural products following a primary screening. Stable HTC/*AvBD9*-*luc* luciferase reporter cells were stimulated with 20 μg/ml of natural products for 24 h in 96-well plates, followed by the luciferase assay. The alamarBlue Dye was added 4 h before the luciferase assay to measure cell viability. The luciferase activity of each compound was normalized to cell viability before the Z-score was calculated.

To identify natural small-molecule compounds with the ability to induce *AvBD9*, natural product and rare natural product libraries of 584 compounds were screened at a final concentration of 20 μg/ml in 96-well plates. The Z-scores of all compounds tested were shown in Figure 3. Using a Z-score of 2.0 as the threshold (Curtis et al., 2016), 21 hits were identified and they represent a structurally diverse group of natural products, with a majority being flavonoids and alkaloids (Table 2). To our surprise, many compounds are involved in epigenetics by regulating histone modification and DNA repair (Table 2). It is noted that none of the compounds had a Z-score of less than −2.0, suggesting that none had a strong activity to suppress *AvBD9* gene expression.

**Table 2:** The Z-scores and major functions of 21 hits at 20 μg/ml from primary screening of the Natural Products Library.

**Compound name**	**CAS number**	**Mass (g/mol)**	**Z score**	**Structural family**	**Major function^a^^,^^b^**
Sanguinarine	5578-73-4	332.1	7.92	Benzophenanthridine alkaloid	Inducer of DNA damage
Datiscetin	480-15-9	286.2	7.66	Hydroxylated flavonoid	?
Wortmannin	19545-26-7	428.4	5.86	Steroid	Inhibitor of PI3K/DNA-PK and DNA repair
HC toxin	83209-65-8	436.5	5.55	Cyclic tetrapeptide	HDAC inhibitor
Hypocrellin B	123940-54-5	528.5	5.18	Perylenequinone	Inducer of DNA strand breakage
Parthenolide	20554-84-1	248.3	4.99	Sesquiterpene lactone	HDAC inhibitor
Tetrandrine	518-34-3	622.8	4.88	Bisbenzylisoquinoline alkaloid	Calcium channel blocker
Apicidin	183506-66-3	623.8	4.42	Cyclic tetrapeptide	HDAC inhibitor
(–)-Depudecin	139508-73-9	212.2	3.95	Polyketide	HDAC inhibitor
Isotetrandrine	477-57-6	622.8	3.91	Bisbenzylisoquinoline alkaloid	Calcium channel blocker
Silibinin	22888-70-6	482.4	3.11	Flavonolignan	STAT3, cyclo- and lipoxygenase inhibitor
Sclerotiorin	549-23-5	390.9	3.1	Azaphilone	HSP90 and lipoxygenase inhibitor
Cytochalasin D	22144-77-0	507.6	3.04	Alkaloid	Actin polymerization inhibitor
Trichostatin A	58880-19-6	302.4	2.7	Hydroxamic acid	HDAC inhibitor
Radicicol	12772-57-5	364.8	2.31	Polyketide	HSP90 and DNA topoisomerase II inhibitor
Tamarixetin	603-61-2	316.3	2.29	O-methylated flavonoid	?
Carminic acid	1260-17-9	492.4	2.07	Glucosidal hydroxyanthrapurin	?
Forskolin	66575-29-9	410.5	2.06	Labdane diterpene	Adenylyl cyclase agonist
Dihydroergocristine mesylate	24730-10-7	707.8	2.06	Ergot alkaloid	Serotonin receptor antagonist
Brassinin	105748-59-2	236.4	2.04	Indole phytoalexin	STAT3 and PI3K/Akt/mTOR inhibitor
Robinetin	490-31-3	302.2	2.01	Hydroxylated flavonoid	?

a *Although there is little information on their biological activities, datiscetin, tamarixetin, and robinetin are expected to have similar epigenetic functions in histone acetylation to structurally related quercetin*.

b *PI3K, phosphatidylinositide 3-kinases; DNA-PK, DNA-dependent protein kinase; HDAC, histone deacetylase; STAT3, signal transducer and activator of transcription 3; HSP90, heat shock protein 90; AKT, protein kinase B; mTOR, mechanistic target of rapamycin*.

**Figure 4:**
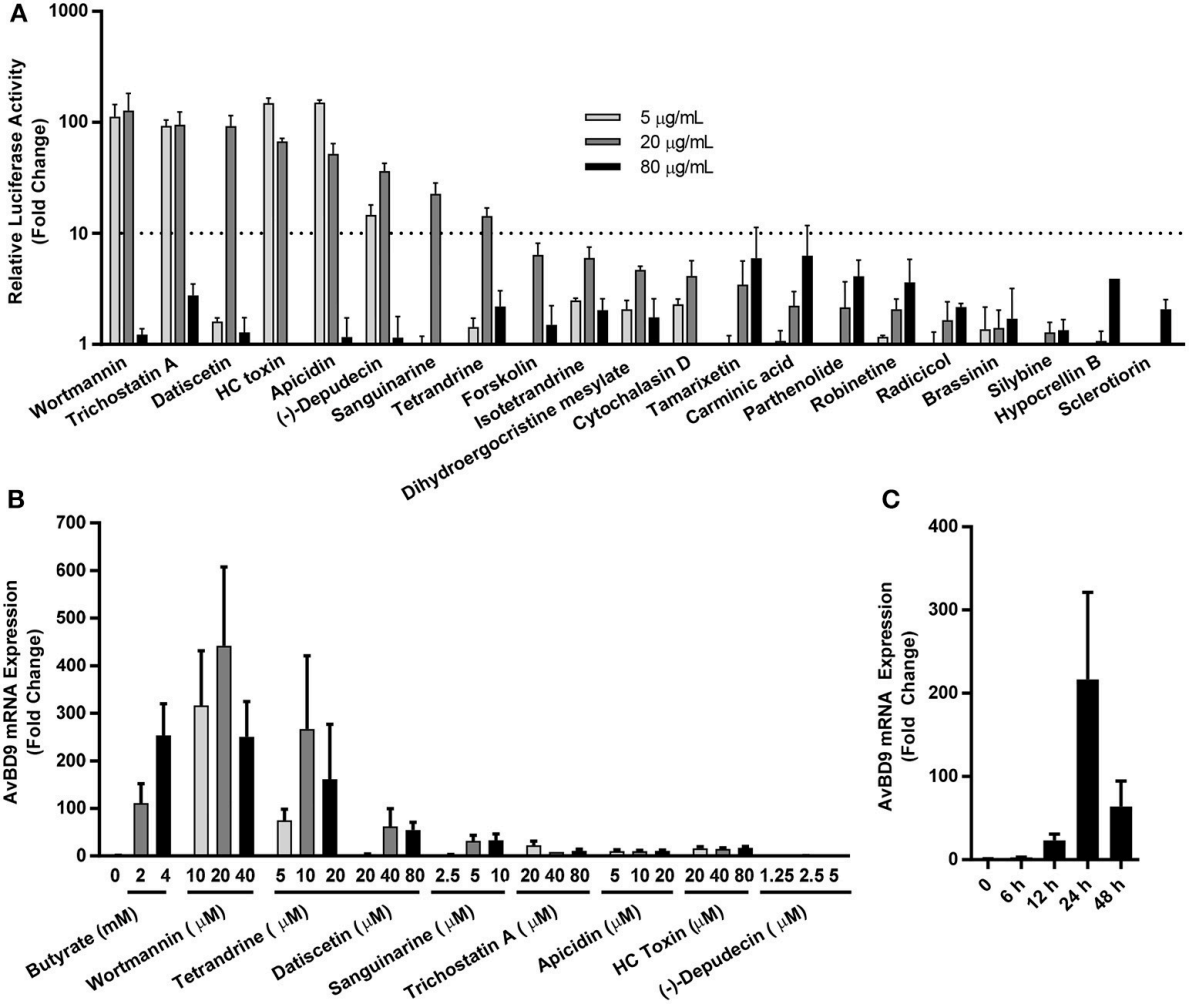
Secondary screening of newly identified natural HDP-inducing compounds. **(A)** Dose-dependent changes in luciferase activity in stable HTC/*AvBD9*-*luc* luciferase reporter cells in response to 21 hits identified in the primary screening. **(B)** Dose-dependent induction of *AvBD9* mRNA expression in parental HTC cells stimulated with selected compounds for 24 h by RT-qPCR. **(C)** Time-dependent changes in *AvBD9* mRNA expression levels in HTC cells treated with 10 μM wortmannin by RT-qPCR. The results are means ± SEM of three independent experiments.

To further validate the *AvBD9*-inducing activity of the 21 hits identified in the primary screening, dose-response experiments were conducted in stable HTC/*AvBD9-luc* cells. When applied at 5, 20, and 80 μg/ml for 24 h, all compounds showed an obvious dose-dependent change in luciferase activity, and at least one concentration of each compound resulted in an increased luciferase activity (Figure 4A), indicative of the validity of our primary screening. Out of 21 compounds, eight including datiscetin, wortmannin, tetrandrine, trichostatin A, HC toxin, (–)-depudecin, apicidin, and sanguinarine had a higher than 10-fold increase in luciferase activity, and were consequently chosen for further confirmation of mRNA expression in parental HTC cells by RT-qPCR. As expected, all compounds, except for (–)-depudecin, induced *AvBD9* mRNA expression (Figure 4B), signifying the reliability of the HTS assay in identifying *AvBD9*-inducing compounds. It is currently unknown why (–)-depudecin, a known HDP inducer in human cells (Kallsen et al., 2012), purchased from two different vendors including BioVision (Milpitas, CA) and MyBioSource (San Diego, CA) failed to work. It is likely because of a variation in structural integrity among difference sources as (–)-depudecin is chemically instable due to the presence of two oxirane rings separated by a *trans* double bond (Kwon et al., 1998).

Among the compounds that induced *AvBD9* mRNA expression, wortmannin, tetrandrine, datiscetin, and sanguinarine were the most potent (Figure 4B). Wortmannin and tetrandrine, when used in the μM range, showed a comparable, if not superior, fold increase to 2 or 4 mM butyrate that gave a maximum 100- to 250-fold *AvBD9* mRNA induction. We further confirmed in a time-course experiment that wortmannin gave a peak induction of *AvBD9* mRNA expression at 24 h (Figure 4C). It is worth mentioning that higher doses of most compounds showed diminished *AvBD9* induction, suggesting the existence of a negative feedback mechanism.

### *Ex vivo* and *in vivo* confirmation of *AvBD9* induction

**Figure 5:**
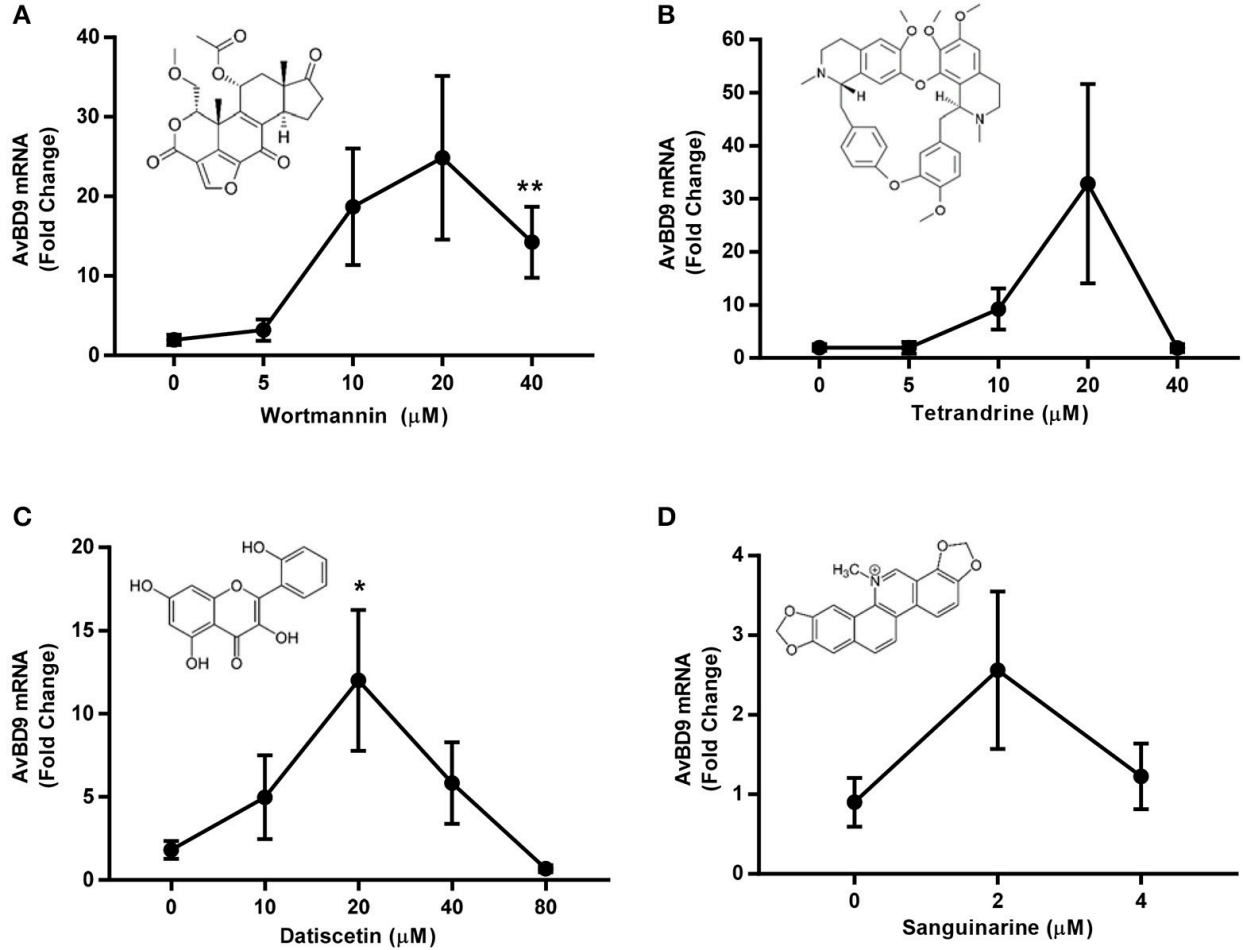
Dose-dependent induction of *AvBD9* mRNA expression in response to wortmannin, tetrandrine, datiscetin, and sanguinarine in chicken jejunal explants. Jejunal explants were prepared and treated with different concentrations of each compound for 24 h, followed by RT-qPCR analysis of *AvBD9* mRNA expression. The results are means ± SEM of 2–3 independent experiments. ^*^P < 0.05 and ^**^P < 0.01 (by unpaired Student's *t*-test).

**Figure 6:**
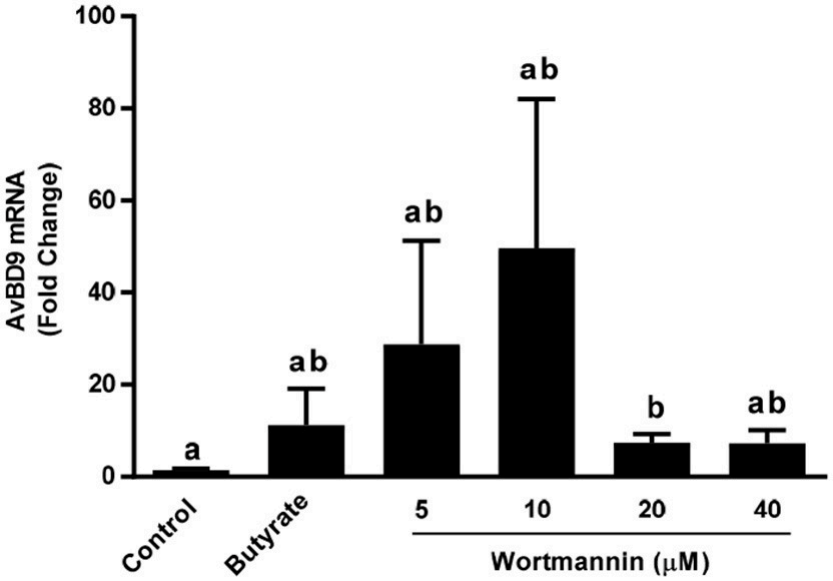
*In vivo* induction of *AvBD9* mRNA expression in the duodenum of chickens by wortmannin. Different concentrations of wortmannin or 40 mM sodium butyrate were administered to 3-day-old chickens (*n* = 12) by oral gavage every 12 h for 36 h. A segment of the mid-duodenum was collected and subjected to total RNA isolation and RT-qPCR analysis of *AvBD9* mRNA expression. Fold changes were calculated relative to the control chickens receiving an equal volume of saline three times. The bars without common superscript letters denote statistical significance (by unpaired Student's *t*-test).

To verify the ability of individual compounds to induce *AvBD9* expression in the intestinal tract, chicken jejunal explants were prepared and stimulated with different concentrations of wortmannin, tetrandrine, datiscetin, and sanguinarine for 24 h. All four compounds showed an obvious dose-dependent induction of *AvBD9* in jejunal explants. The optimal dose for wortmannin, tetrandrine, and datiscetin was 20 μM with a 15- to 40-fold induction of *AvBD9* mRNA, while 2 μM sanguinarine gave a peak induction of approximately 2.5-fold (Figure 5). To further confirm whether wortmannin is capable of inducing *AvBD9 in vivo*, 3-day-old broiler chickens were given 5, 10, 20, or 40 μM wortmannin or 40 mM butyrate by oral gavage every 12 h for 36 h. RT-qPCR analysis of *AvBD9* gene expression in the duodenum revealed that 5 and 10 μM wortmannin increased *AvBD9* mRNA expression by approximately 30- and 50-fold, respectively, which was superior to 40 mM butyrate showing a 10-fold increase (Figure 6).

### Induction of multiple chicken HDP genes by natural compounds and their synergy with butyrate

**Figure 7:**
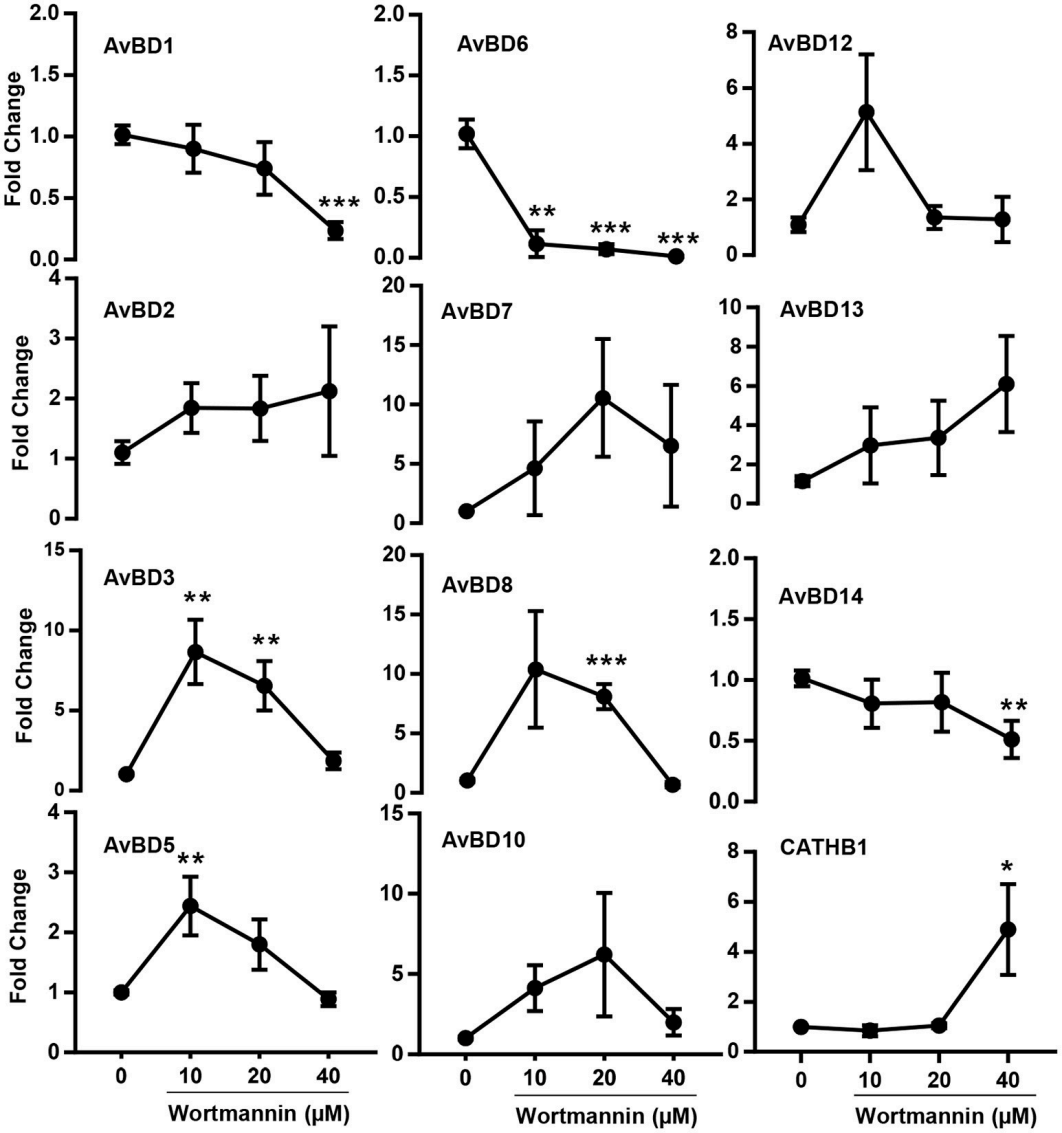
Dose-dependent changes in multiple chicken HDP mRNA expression levels in HTC cells by wortmannin. Chicken HTC cells were treated with or without three different concentrations of wortmannin for 24 h, followed by RT-qPCR analysis of mRNA expression of all chicken HDP genes that can be detected in HTC cells. The results are means ± SEM of three independent experiments. ^*^P < 0.05, ^**^P < 0.01 and ^***^P < 0.001 (by unpaired Student's *t*-test).

Besides *AvBD9*, 13 other β-defensins and 4 cathelicidins exist in chickens (Cuperus et al., 2013; Zhang and Sunkara, 2014) and butyrate can induce more than a half number of them (Sunkara et al., 2011). To examine how other chicken HDP genes are regulated by wortmannin, HTC cells were stimulated with or without three different doses (10, 20, and 40 μM) of wortmannin for 24 h, followed by RT-qPCR of individual β-defensins and cathelicidins. Among those HDP genes that can be detected in HTC cells, all but three were obviously induced by wortmannin, albeit with a reduced magnitude of induction relative to *AvBD9* (Figure 7). Clearly, different HDP gene showed different patterns of induction. Wortmannin dose-dependently increased *AvBD2, AvBD13*, and *CATHB1* gene expression, while *AvBD3, 5, 7, 8, 9, 10*, and *12* had a peak induction at 10 or 20 μM (Figure 7). On the other hand, *AvBD1, 6*, and *14* was dose-dependently suppressed by wortmannin.

**Figure 8:**
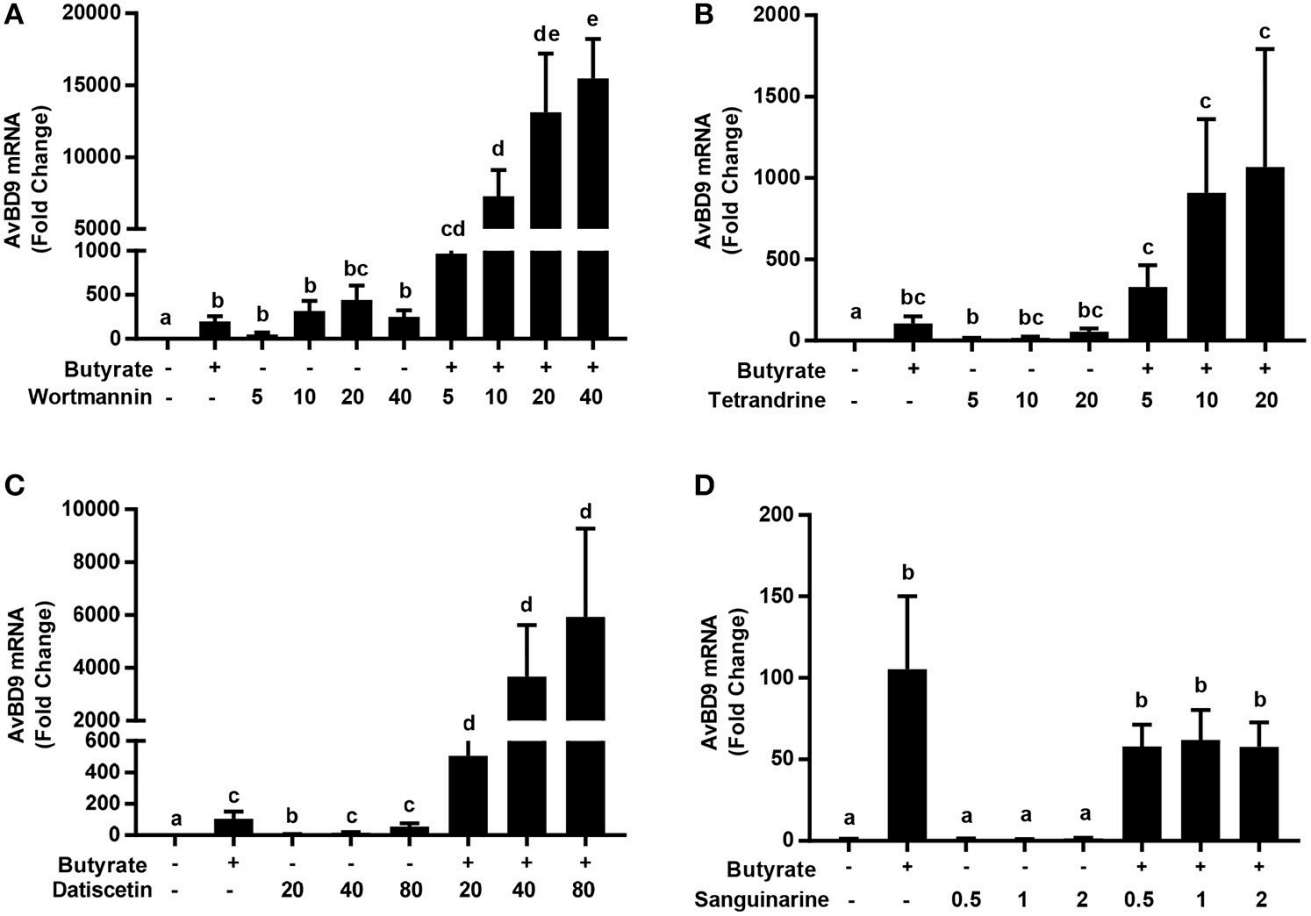
Synergistic induction of *AvBD9* mRNA expression in HTC cells between butyrate and three newly identified natural products. Chicken HTC cells were treated with 4 mM butyrate in the presence or absence of different concentrations of wortmannin **(A)**, tetrandrine **(B)**, datiscetin **(C)**, or sanguinarine **(D)** for 24 h, followed by RT-qPCR analysis of *AvBD9* mRNA expression. The results are means ± SEM of 2–3 independent experiments. The bars without common superscript letters denote statistical significance (by unpaired Student's *t*-test). It is noted that an obvious synergy in AvBD9 gene expression was observed between butyrate and any of wortmannin, tetrandrine, datiscetin, but not between butyrate and sanguinarine.

Intrigued by the synergy between butyrate with other small-molecule compounds such as vitamin D_3_ (Schauber et al., 2008), lactose (Cederlund et al., 2013), and forskolin (Sunkara et al., 2014), we sought to evaluate a possible synergistic action between butyrate and several newly-identified HDP-inducing compounds. A dramatic synergy was observed between butyrate and any of wortmannin, tetrandrine and datiscetin in HTC cells, but not between butyrate and sanguinarine (Figures 8A–D). For example, 4 mM butyrate and 40 μM wortmannin individually enhanced *AvBD9* expression by approximately 200- and 250-fold, respectively, while a combination of 4 mM butyrate and 40 μM wortmannin induced *AvBD9* expression by approximately 15,500-fold, which reflected an additional 60-fold increase over either compound alone (Figure 8A). Similarly, the butyrate/tetrandrine (Figure 8B) and butyrate/datiscetin combinations (Figure 8C) also displayed a strong synergy separately. However, no synergy was observed between butyrate and sanguinarine (Figure 8C), suggesting the mechanisms of action among different compounds are likely to be different.

### Augmentation of the antibacterial activity of chicken monocytes by wortmannin

**Figure 9:**
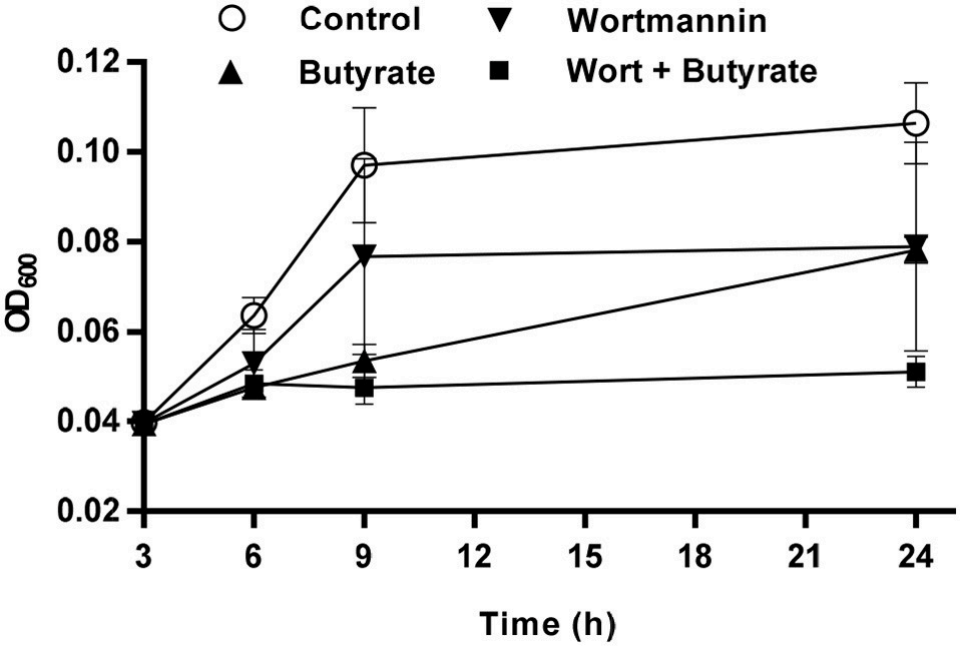
Augmentation of the antibacterial activity of chicken monocytes by wortmannin and butyrate. Chicken monocytes were stimulated with 40 μM wortmannin, 4 mM butyrate or their combination for 24 h. Cell lysates were then incubated with *Salmonella enteritidis* (ATCC 13076) at 37°C. The bacterial turbidity was measured at OD_600_ at 3, 6, 9, and 24 h. The results are means ± SEM of two independent experiments.

HDP inducers such as butyrate and vitamin D_3_ are capable of enhancing the antibacterial activity of host cells (Schauber et al., 2006, 2008; Liu et al., 2007; Sunkara et al., 2011; Rekha et al., 2015) and alleviate disease symptoms (Sarker et al., 2011; Zhao et al., 2014; Mily et al., 2015). To confirm whether wortmannin or the combination of wortmannin and butyrate can augment the antibacterial activity of host cells, chicken monocytes were isolated and stimulated with 40 μM wortmannin, 4 mM sodium butyrate or their combination for 24 h, followed by incubation of the cell lysate with *S. enteritidis* (ATCC 13076) and measurement of the bacterial turbidity (Schauber et al., 2006; Sunkara et al., 2011). Consistent with our earlier observation (Sunkara et al., 2011), butyrate-treated monocytes exhibited an obviously enhanced ability to suppress bacterial growth (Figure 9). Wortmannin also improved the ability of monocytes to kill bacteria. Importantly, a combination of wortmannin and butyrate resulted in nearly complete suppression of bacterial growth up to 24 h, suggestive of their synergistic activity.

To rule out the possibility that augmented bacterial killing of chicken monocytes is due to direct antibacterial activity of wortmannin or butyrate, a standard broth microdilution assay (National Committee for Clinical Laboratory Standards, 2003) was performed using two reference bacterial strains, *S. enteritidis* (ATCC 13076) and *E. coli* (ATCC 25922) exposed to wortmannin in serial 2-fold dilutions in the presence or absence of 4 mM butyrate. Wortmannin alone or in combination with 4 mM butyrate showed no obvious antibacterial activity, with the MIC beyond 320 μM, the highest concentration that we tested (data not shown), implying that wortmannin, particularly the wortmannin/butyrate combination, could enhance HDP synthesis and bacterial clearance without exerting selective pressure on bacteria, thus reducing the likelihood of triggering bacterial resistance against HDP inducers.

### Involvement of the PI3K/AKT/mTOR pathway in *AvBD9* gene induction

**Figure 10:**
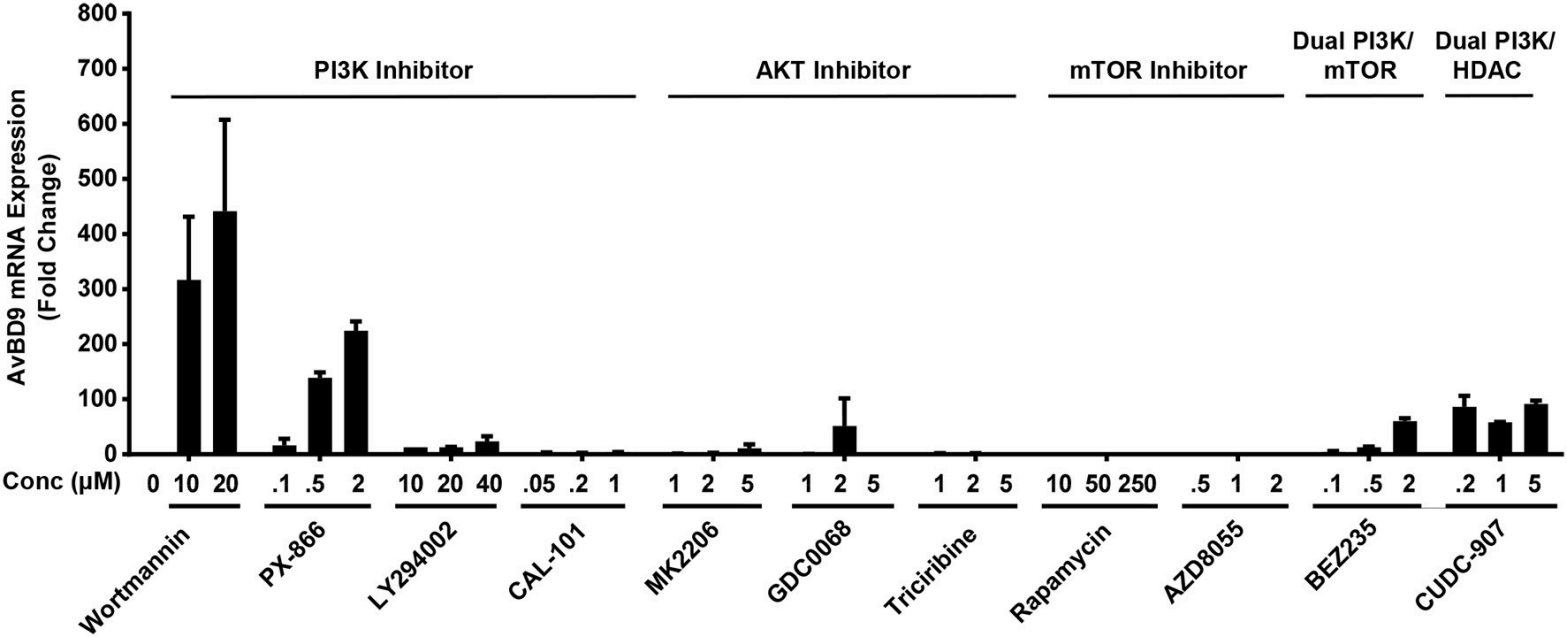
Involvement of the PI3K/AKT/mTOR signaling pathway in AvBD9 mRNA induction. Chicken HTC cells were treated with or without different concentrations of specific inhibitors to PI3K (Wortmannin, PX-866, LY294002, and CAL-101), AKT (MK2206, GDC0068, triciribine) or mTOR (Rapamycin, AZD8055), dual PI3K/mTOR inhibitor (BEZ235) or dual PI3K/HDAC inhibitor (CUDC-907) for 24 h, followed by RT-qPCR analysis of *AvBD9* gene expression. The results are means ± SEM of 2–3 independent experiments.

Phosphoinositide 3-kinases (PI3K) are a family of structurally related enzymes that are involved in a variety of cellular functions that often signal through protein kinase B (also known as AKT) and mechanistic target of rapamycin (mTOR) (Polivka and Janku, 2014; Fruman et al., 2017). Wortmannin is a well-known inhibitor of PI3K (Ui et al., 1995) and brassinin is a newly identified AvBD9-inducing compound (Table 2) also with a reported PI3K inhibitory activity (Izutani et al., 2012). To examine whether the PI3K/AKT/mTOR pathway is involved in *AvBD9* gene induction, specific inhibitors to PI3K (PX-866, LY294002, and CAL-101), AKT (MK2206, GDC0068, triciribine) or mTOR (Rapamycin, AZD8055) or dual inhibitors to PI3K/mTOR (BEZ235) or PI3K/HDAC (CUDC-907) were applied to HTC cells separately for 24 h, followed by RT-qPCR analysis of *AvBD9* gene expression. Among all four PI3K inhibitors, only pan-inhibitors, wortmannin and its structural analog PX-866, gave a robust *AvBD9* induction, while another pan-inhibitor (LY294002) and an isoform-specific inhibitor (CAL-101) showed a minimum or no activity (Figure 10), suggesting that specific inhibition of PI3K may have a limited effect on *AvBD9* induction. The reason that wortmannin and PX-866 work well is likely due to their non-specific activities. Wortmannin is highly efficient in suppressing PI3K in the low nanomolar range, but can non-specifically inhibit several other PI3K-related kinases such as DNA-dependent protein kinase (DNA-PK) at higher concentrations (Wymann et al., 1996). In this study, the micromolar concentrations of wortmannin are needed to induce chicken HDP genes, and no appreciable HDP gene induction was observed when wortmannin was used below 1 μM (data not shown). Therefore, it is likely that PI3K inhibition alone is insufficient for robust HDP gene induction. In agreement, none of the three AKT inhibitors or mTOR inhibitors had an obvious ability to induce *AvBD9* expression (Figure 10), suggesting a minimum involvement of the PKA/AKT/mTOR signaling pathway in chicken *AvBD9* induction. Interestingly, dual inhibition of PI3K/mTOR or PI3K/HDACs gave obvious *AvBD9* expression in chicken HTC cells, albeit with a much reduced fold increase relative to wortmannin or PX-866 (Figure 10).

## Discussion

Increased resistance to conventional antibiotics necessitates the development of novel antimicrobial strategies. With no or reduced likelihood of triggering resistance, host-directed antimicrobial therapies are gaining increased attention, with a number of products being approved for human use or evaluated at different stages of clinical trials (Zumla et al., 2016). Moderating the synthesis of endogenous HDPs has shown promise in reducing infections and alleviating clinical infections and is being actively explored as an alternative approach to antimicrobial therapy (Campbell et al., 2012; Van Der Does et al., 2012; Lyu et al., 2015). A number of small-molecule compounds such as butyrate and vitamin D_3_ have been identified to induce HDP synthesis in humans and other animal species (Campbell et al., 2012; Van Der Does et al., 2012; Lyu et al., 2015). A cell-based HTS assay was recently developed by employing human HT-29 intestinal epithelial cells transfected with a fusion of a 4-Kb human cathelicidin *LL-37* gene promoter and its entire open reading frame with a luciferase reporter gene (Nylen et al., 2014). Such an approach has led to discovery of a group of compounds with the ability to induce *LL-37* gene expression; however, a majority of the compounds are weak relative to many known LL-37 inducers such as butyrate (Nylen et al., 2014). To facilitate the identification of additional, perhaps more potent HDP inducers particularly for poultry applications, we developed a HTS assay by fusing a luciferase gene with a 2-Kb *AvBD9* gene promoter, followed by lentiviral transduction into a chicken macrophage cell line. After optimization, we obtained a Z'-factor of 0.80 for our HTS assay, which suggested that it is a robust system (Zhang et al., 1999) and equivalent to the previously reported human HTS assay that had a Z'-factor of approximately 0.70 (Nylen et al., 2014). By employing such a HTS assay, we identified 21 natural compounds with a strong ability to boost *AvBD9* gene expression after a screening of 584 natural products.

Out of the 21 HDP-inducing compounds identified in this study, only forskolin, trichostatin A (TSA), apicidin, and (–)-depudecin have been reported earlier with the ability to induce HDP gene expression in humans and chickens (Yin and Chung, 2011; Kallsen et al., 2012; Sunkara et al., 2014). The 17 remaining compounds are linked with a role in HDP induction for the first time. To our surprise, 14 out of 21 HDP-inducing compounds are involved in epigenetic modifications of DNA, histones or non-histone proteins (Table 2). TSA, HC toxin, parthenolide, apicidin, and (–)-depudecin are known histone deacetylase (HDAC) inhibitors (Bassett and Barnett, 2014), which contribute to histone hyper-acetylation, chromosomal relaxation, and often enhanced gene expression (Chen et al., 2015). Consistently, human HDPs such as LL-37, β-defensin-1, and β-defensin-2 have been found to be upregulated by HDAC inhibitors such as butyrate, TSA, apicidin, sulforaphane, curcumin, MS-275, and resveratrol and its analogs (Yedery and Jerse, 2015). Although it has not been definitively confirmed, datiscetin, tamarixetin, and robinetin are all structurally similar to quercetin, a natural flavonol with the ability to regulate the activities of histone acetyltransferases, sirtuins, and classical HDACs (Lee et al., 2011; Xiao et al., 2011; Trevino-Saldana and Garcia-Rivas, 2017). Therefore, it is very likely these quercetin-like flavonoids have histone modifying functions as well. Besides histone acetylation, four compounds including sanguinarine, wortmannin, hypocrellin B, and radicicol are known to induce DNA damage, inhibit DNA repair or DNA topoisomerase II activity (Xu et al., 1998; Hashimoto et al., 2003; Barker et al., 2006; Matkar et al., 2008), which has been recently revealed to exert a positive role in initiation of gene transcription (Pommier et al., 2016; Vitelli et al., 2017). Additionally, radicicol, sclerotiorin, and sanguinarine are capable of inhibiting heat shock protein 90 (HSP90) (Davenport et al., 2014; Kabbaj et al., 2015; Khandelwal et al., 2016), which is known to interact with DNA topoisomerase II (Barker et al., 2006) and whose activity is regulated by reversible acetylation (Prodromou, 2016). Thus, inhibition of DNA repair or HSP90 could positively impact the transcription of a subset of genes perhaps including many HDPs.

Wortmannin, tetrandrine, datiscetin, and sanguinarine are among the four most potent *AvBD9*-inducing compounds identified in this study. Wortmannin is a fungal metabolite and a well-known inhibitor of phosphoinositide 3-kinases (PI3K) (Ui et al., 1995), which are critically involved in a variety of cellular metabolism and immune functions (Fruman et al., 2017). However, the PI3K/AKT/mTOR pathway alone appears to play a minimum role in *AvBD9* gene induction because most specific inhibitors to PI3K, AKT and mTOR are largely ineffective in inducing *AvBD9*. The reason that wortmannin is highly efficient is likely due to its dual inhibitory role to both PI3K and DNA-PK (Hashimoto et al., 2003), with the latter being required to repair double-strand DNA breaks via the non-homologous end joining pathway (Davis et al., 2014; Blackford and Jackson, 2017). Inhibition of both PI3K and DNA repair perhaps creates a synergistic effect on HDP gene induction.

Among the other three potent *AvBD9* inducers, tetrandrine is a *bis*-benzylisoquinoline alkaloid extracted from the root of a Chinese herb, *Stephania tetrandra* S. Moore (Bhagya and Chandrashekar, 2016; Liu et al., 2016). Datiscetin is a plant-derived flavonoid and structurally related to quercetin that is known to have epigenetic functions (Lau and Chang, 2015) and a strong ability to induce chicken HDP genes (data not shown). Therefore, it is of little surprise that datiscetin is capable of inducing *AvBD9*. Sanguinarine is a benzophenanthridine alkaloid extracted from the bloodroot plant *Sanguinaria canadensis* (Selvi et al., 2009) that can cause DNA damage (Matkar et al., 2008), which could subsequently lead to an increase in *AvBD9* gene transcription. However, the mechanism by which tetrandrine induces AvBD9 expression remains unknown. Tetrandrine is a well-known calcium channel blocker and has been used as a Chinese traditional medicine for decades to treat hypertensive and arrhythmic conditions, inflammation, fibrosis, and silicosis (Bhagya and Chandrashekar, 2016; Liu et al., 2016). Whether tetrandrine augments *AvBD9* expression by acting as a calcium channel blocker warrants further investigations. We observed a strong synergy in *AvBD9* gene induction between butyrate, a well-studied pan-HDAC inhibitor, and any of wortmannin, datiscetin and tetrandrine, but not between butyrate and sanguinarine. The mechanism behind their synergy needs to be further investigated.

Although structurally divergent from each other, a common feature among wortmannin, tetrandrine, and sanguinarine is that they are all anti-inflammatory, antioxidative, anti-proliferative, and pro-apoptotic (Ui et al., 1995; Selvi et al., 2009; Bhagya and Chandrashekar, 2016; Liu et al., 2016). So is likely the case with datiscetin because of the anti-inflammatory and antioxidative properties associated with structurally-related quercetin (Chirumbolo, 2010). The ability to enhance HDP gene expression and antioxidative response without triggering inflammation makes these compounds desirable for further development as host-directed therapeutics for disease control and prevention. The fact that some of these compounds have demonstrated synergistic actions among each other suggests the potential of employing these compounds or their combinations as alternatives to antibiotics for poultry applications. Additionally, due to a lack of direct antimicrobial activities, these HDP inducers augment host immunity and disease resistance with a minimum risk of triggering resistance. Furthermore, because many HDP inducers have been found to promote HDP synthesis across animal species (Yedery and Jerse, 2015), it is likely that a few, if not all, of these newly-identified compounds are capable of enhancing HDP synthesis and disease control and prevention beyond chickens.

## Author contributions

GZ and WL: conceived and designed the experiments; WL, ZD, LS, SB, and KR: performed the experiments; WL and GZ: analyzed and interpreted the data; RM: provided the reagents; WL and GZ: drafted and revised the manuscript.

### Conflict of interest statement

The authors declare that the research was conducted in the absence of any commercial or financial relationships that could be construed as a potential conflict of interest.
